# Queries Regarding Local Erythropoietin Injection in Tibiofibular Fracture Healing

**DOI:** 10.5812/traumamon.12192

**Published:** 2013-08-19

**Authors:** Jan Hendrik Duedal Rölfing, Cody Bünger

**Affiliations:** 1 Orthopaedic Research Laboratory and Department of Orthopaedics, Aarhus University Hospital, Aarhus, Denmark

**Keywords:** Erythropoietin, Fracture Healing, Bone and Bones, Orthopedics, Orthopedic Procedures, Tibia

*Dear Editor,*

We would like to congratulate Dr. Bakhshi and colleagues for being the first group to publish scientific evidence regarding the osteogenic effect of erythropoietin (EPO) in a clinical trial (1). As you correctly report, several preclinical studies have shown the efficacy of Erythropoietin to increase bone healing at early time points (2-4).In agreement with your approach, we strongly believe that localized, low-dose administration is the prerequisite for clinical application of recombinant human EPO in the clinical orthopedic setting. Systemic dosage increases risk of known adverse effects such as thromboembolic complications.However, the scientific validity of your study could have been greatly improved if the following steps were followed: first, adherence to CONSORT guidelines regarding randomized clinical trials; especially information about eligibility, enrolment, randomization and blinding procedures should be given; secondly, the treatment regimen, e.g. manufacturer and dosage of EPO specified in more detail (5). For instance, the treatment regimen was described as “EPO (4000 IU) was injected in one group into the fracture site two weeks after the operation and under sterile conditions and guide of a C-arm.” However, after email correspondence, the authors specify that they used three vials in slim patients and up to five vials in obese patients. Tibia fractures span a broad range of injuries. Fracture healing and the likelihood of non-unions highly depends upon the severity of the fracture and soft-tissue injury (6). Excellent classification systems for fracture types and accompanying soft-tissue injury exist and should be used when reporting fracture studies (closed fractures: Oestern and Tscherne, open fractures: Gustilo) (6-8). Were fractures in this study confined to the simple tibia shaft fractures with addition of fibular fracture, corresponding to AO classification types: 42-A1.2, 42-A1.3, 42-A2.2, 42-A2.3, 42-A3.2, and 42-A3.3 (9)? Was there any difference in soft-tissue damage between the treatment and control group? Even though Goldhan et al. suggest multiple radiologic examinations in order to determine possible acceleration of fracture healing, in the light of patient safety concerns, 10 radiologic examinations per patient with 14-day intervals until union was achieved at a mean time of almost 20 weeks seem clinically and ethically unjustified (10)
